# Threats of Longline Fishing to Global Albatross Diversity

**DOI:** 10.3390/ani12070887

**Published:** 2022-03-31

**Authors:** Gohar A. Petrossian, Stephen F. Pires, Monique Sosnowski, Prabha Venu, George Olah

**Affiliations:** 1 Department of Criminal Justice, John Jay College of Criminal Justice, 524 West 59th Street, New York, NY 10019, USA; msosnowski@jjay.cuny.edu; 2 Department of Criminology & Criminal Justice, Florida International University, Miami, FL 33199, USA; sfpires@fiu.edu; 3 Independent Researcher, Santa Cruz, CA 95065, USA; pvenu@pobox.com; 4 Fenner School of Environment & Society, The Australian National University, Canberra, ACT 2600, Australia; george.olah@anu.edu.au

**Keywords:** longline fishing, IUU, bycatch, policy, spatial econometrics, GIS, Diomedeidae

## Abstract

**Simple Summary:**

This research examines the impact potential illegal longline fishing vessels have on albatrosses. Using environmental criminology as a guiding theoretical framework, this research: (a) examines the patterns of concentration of potentially illegal longline fishing efforts and their relationships with the spatial distributions of commercially sought-out and illegally-caught fish species; and (b) examines how their interactions affect the average risk of albatrosses. The results indicate that: (a) potentially illegal longline fishing activities are highly spatially concentrated in areas with the highest concentration of the presence of known illegally-caught fish species; and (b) the average risk score of albatrosses is significantly higher in areas where these illegal longline fishing vessels operate. These findings provide strong grounding that illegal longline fishing poses a particularly serious threat to the survival of seabirds. These findings also call for the bird conservation lobby to work closely with regional fisheries management organizations to devise and implement targeted interventions.

**Abstract:**

Albatrosses are among the most threatened seabird species. Often entangled in gillnets or hooked while longline fishing gear is being set, albatrosses are affected by fishing. This is assumed to be especially true in cases where illegal longline fishing vessels are involved, as they are less likely to implement the bycatch mitigation measures implemented to reduce the risk of albatrosses being caught on their hooks. This is the assumption that was tested in the current study, which uses environmental criminology as its guiding theoretical framework. Using the spatial units of one-half-degree by one-half-degree longitude/latitude cells, this research examined the patterns of concentration of potentially illegal longlining efforts and their relationships to commercially sought-out and illegally caught (i.e., CRAAVED—concealable, removable, abundant, accessible, valuable, enjoyable, disposable) fish species concentrations, as well as their effects on the average risk of albatrosses. The results indicated that (a) potentially illegal longlining activity is spatially concentrated; (b) this concentration is exhibited in areas with the highest concentrations of the presence of CRAAVED fish; and (c) the average risk score of albatrosses, as measured by their International Union for Conservation of Nature (IUCN) Red List status, is significantly higher in the areas where illegal longlining vessels are found controlling for the activities of legal longlining vessels. These findings provide strong grounding that illegal longline fishing poses a particularly serious threat to the survival of albatrosses. These activities, however, are not randomly spread across the vast oceans, but rather are highly spatially concentrated. Therefore, the bird conservation lobby should work closely with regional fisheries management organizations to devise and implement targeted interventions aimed at reducing potential illegal longline fishing, which, in turn, will likely have positive effects on albatrosses.

## 1. Introduction

In the early 1950s, commercial, distant-water, pelagic longline fleets significantly expanded their overseas fishing operations for highly commercial tuna (*Thunnini*) and tuna-like species [1,2]. The increase in these activities was soon accompanied by a significant conservation concern for seabirds [3], as longline fishing was often accompanied by the problem of bycatch, or the incidental catch of non-target species [4]. While there are many seabird species that are frequently caught by longliners, such as northern fulmar (*Fulmarus glacialis*) and various gull species, albatrosses are disproportionately affected by longline operations. Of the 61 species of seabirds harmed by longline fishing, 26 are threatened with extinction [5], of which 17 are albatrosses [6]. These 17 represent over 77% of all albatross species and, therefore, of all birds, albatrosses are the most threatened by longline fishing.

Albatrosses (Diomedeidae) are large, long-lived, and iconic seabirds, currently comprising 22 extant species distributed in the Southern Ocean and North Pacific. They are incidentally caught during demersal and pelagic longline fishing operations [1]. They die after being caught on baited hooks set to catch tuna, Patagonian toothfish (*Dissostichus eleginoides*) and other finfish species while gear is being set, and are subsequently dragged under water, where they drown [7,8]. In fact, increased longline fishing efforts are associated with increased bycatch rates of not only seabirds, but also marine species, such as sea turtles, sharks, and dolphins [9].

The association between longline fishing and seabird mortality has been established in many studies conducted at the international, regional, and country levels (e.g., [4,10,11,12,13]). However, for all the attention directed at investigating the association between longline fishing and seabird mortality, important research gaps remain. More specifically, little attention has been directed at investigating whether potentially illegal longline fishing also has adverse impacts on seabird populations. Illegal longline vessels flaunt the regulations set forth by regional fisheries management organizations or other international maritime regulatory bodies, such as bycatch mitigation measures [14]. Consequently, such blatant disregard for rules and regulations increases the probability that such vessels are increasing albatross bycatch rates.

The lone study that has examined the relationship between illegal longline fishing and albatross extinction risk found that species exposure to illegal fishing on the coastal waters of one nation—undisclosed in the study—was associated with a higher conservation category on the IUCN Red List [15]. Importantly, critically endangered (CR) albatrosses were 12 times and endangered (EN) albatrosses were 3.4 times more exposed to illegal longline hooks than any near-threatened (NT) seabird species. Whether the results from a single nation are generalizable to other countries, or even the rest of the world, has been unexplored.

In this study, we advance research in this area in two ways. First, this study examines the cumulative longline fishing activities (longlining hereafter) of potentially illegal fishing vessels flagged to the 35 known “flags of convenience” (FOC) countries—a proxy measure for illegal longlining activity [16,17,18,19]—to determine their cumulative impact on albatrosses. As a result, this study focuses on global longlining activities and their overlap with the at-sea ranges of all 22 albatross species as opposed to a small geographic region (see [15]). Second, this study will use a much more refined unit of analysis, the one-half-degree by one-half-degree longitude/latitude cell, to study the exposure of albatrosses to FOC-flagged vessels (assumed to have a higher likelihood for fishing illegally). No study to date has examined the impact of illegal longlining on albatrosses at this high-resolution level and at the global scale. Knowing the patterns where albatrosses will be most at risk of being killed by longlines will allow for the implementation of spatially explicit response strategies.

## 2. Materials and Methods

### 2.1. Theoretical Framework

This study draws on the environmental criminology or crime science perspective, which comprises a suite of criminological theories that focus on explaining criminal events in the context of the immediate environment in which these events unfold [20]. Theories within this family focus on explaining the interaction between a motivated offender and the opportunities for committing crimes created by the environment. The most important premise of this perspective is that crime is not randomly distributed but, rather, it is highly concentrated in time and space [21,22] and among offenders and victims [23,24], and that opportunities for crime in the environment also vary over time and space. 

In the past decade, the theories of environmental criminology have been applied to the wildlife crime context in various ways to identify and explain the opportunity structures in the environment that enable such crimes. Its first application was manifested in the Lemieux and Clarke 2009 study of elephant poaching in Africa [25]. Subsequently, researchers have applied these theories to the study of parrot poaching in Latin America [26,27] and Indonesia [28]; illegal commercial fishing [29,30]; illegal recreational fishing [31,32]; tiger poaching [33]; human–leopard conflict [34]; redwood burl poaching [35]; and others. The collective findings of these studies have demonstrated the utility of examining such crimes through the environmental criminological theoretical lens, because evidence-based intervention strategies can then be directly derived from within these theoretical foundations to effectively deal with such problems. 

**Figure 1:**
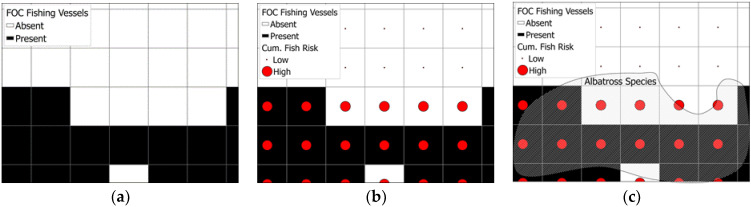
Conceptualizing the relationship among illegal longline fishing, albatrosses, and commercially viable fish. In (**a**), the presence of FOC longliners is visible in the black-colored grid cells. FOC longliners tend to be in areas where there is a greater risk score of commercially viable fish, as noted by the larger graduated symbols in (**b**). In (**c**), the theoretical relationship among all three variables is visible. FOC longliners are present in areas with higher risk scores of commercially viable fish and these same areas tend to overlap with distributions of albatross species.

This study utilizes crime pattern theory [36] to explain how victims and offenders converge in time and space and how the repetitive mobility of the offenders creates the “awareness spaces” within which the offenders feel comfortable to engage in criminal behavior. In light of this theoretical underpinning, this study assumes that albatrosses will be most at risk in areas where their foraging sites overlap with areas with high levels of potentially illegal longline fishing activity. This is because the “activity space” of albatrosses intersect with the “awareness space” of the illegal longline fishers, making albatrosses significantly more vulnerable to bycatch by these vessels. The theory assumes that crime will be highest around the nodes, as well as along the paths and edges (or the boundaries of their activities) of the daily routine that motivated offenders frequent. In this case, it is assumed that the areas with the distribution of albatross species of higher Red List conservation status (in terms of their vulnerability scores) are the areas where illegal longlining activity happens most frequently. Moreover, it is assumed that these areas will be along the paths and edges and within the nodes of fishing activities of the longline vessels flagged to FOC flags. Importantly, the activities of these vessels likely engaged in illegal fishing will predictably be around the areas where the highest concentration of highly sought-after and commercially viable (or CRAAVED) fish (see [37]) can be found (see Figure 1). 

Illegal fishers are far less likely to conform to mitigation measures designed to reduce bycatch, such as bird-scaring devices, underwater settings, and line weighting [15], which are measures that have generally been effective in reducing bycatch [5] and are far more likely to be employed by legal fishing vessels. It is, therefore, important to identify these at-risk areas so that more focused prevention efforts can be developed to protect the at-risk species from becoming bycatch for illegal longline vessels.

### 2.2. Hypotheses

Consistent with one of the most important premises of the environmental criminological theories, we expect to see that potentially illegal longlining activity will be spatially concentrated. Such activity will be clustered because these are the same areas where in-demand or CRAAVED fish are disproportionally found. At the same time, we expect the “activity spaces” of albatrosses to overlap with those of CRAAVED fish, resulting in a convergence in space of potentially illegal longlining activity, CRAAVED fish, and at-risk albatrosses. Deriving from these assumptions, we propose the following hypotheses:

**Hypothesis** **1.** *Potential illegal longline fishing efforts will be concentrated within a relatively small geographic area.*

**Hypothesis** **2.** *Potential illegal longline fishing efforts will be concentrated within the CRAAVED fish risk spaces.*

**Hypothesis** **3.** *When controlling for the presence of likely legal longline fishing activities and other important environmental factors, the average risk of albatrosses (measured in terms of their Red List status) can be explained by the presence of potentially illegal longline fishing vessels and the cumulative risk score of high-risk CRAAVED fish.*

#### 2.2.1. Dependent Variable

**Figure 2:**
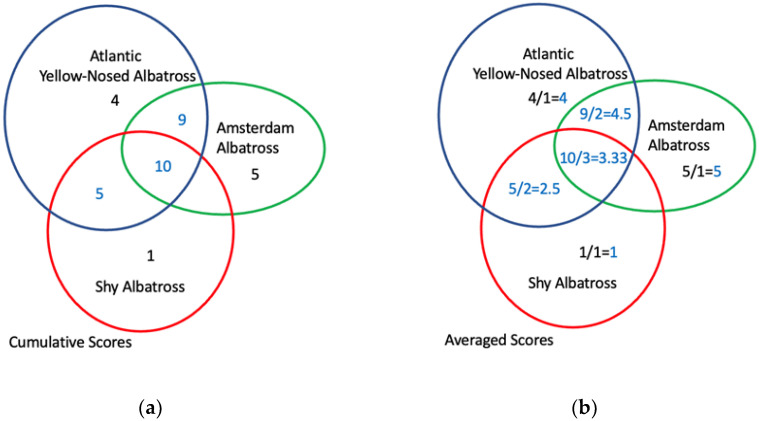
An example of assigning risk scores to grid cells for overlapping areas of albatross at-sea ranges. (**a**) Cumulative Albatross IUCN Red List Scores by Grid Cell; (**b**) Average Albatross IUCN Red List Scores by Grid Cell. The scores were calculated by summing the IUCN Red List status scores (0–5) of the albatross species whose at-sea ranges overlap with a given grid cell divided by the number of albatross species found within these grid cells.

The dependent variable is the average risk score of albatrosses calculated as the sum of the risk score of the albatrosses measured by their IUCN Red List status divided by the number of albatrosses within a given cell. This is conceptualized as a proxy measure of the highest-risk spaces for albatross extinction risk. Similar to Petrossian [30], we first assigned risk scores to each one-half-degree by one-half-degree grid cell that overlapped with the at-sea ranges of each of the 22 albatross species. These risk scores were measured using the cumulative Red List status scores for each of these albatross species. For example, if a grid cell area overlapped with the Amsterdam Albatross *Diomedea amsterdamensis* (CR = 5), Atlantic Yellow-nosed Albatross *Thalassarche chlororhynchos* (EN = 4), and Shy Albatross *T. cauta* (NT = 1) at-sea ranges, the cumulative risk-score for this grid cell would be 11 (as demonstrated in Figure 2). Grid cells that have no albatrosses received a score of “0”. After these calculations were performed for each grid cell in the study area, the final cumulative scores within each albatross-present grid cell were divided by the number of albatrosses present within that cell to standardize the values across all grid cells. For example, the hypothetical cumulative risk score of 11 mentioned above would be coded as 3.67, because there were three species that contributed to that 11 score.

Data on albatross at-sea geographical ranges were obtained from BirdLife International [38]. Additional data, such as albatross species Red List status, were obtained from the International Union for Conservation of Nature [39] from the year 2016 to coincide with the longline fishing data below.

#### 2.2.2. Independent Variable

***FOC Longlining Activity Risk Space*.** A large body of literature [16,17,18,19,40,41,42,43] suggests that many illegal fishing vessels take advantage of the availability of flags of convenience to carry out their activities with little regard to the regulations set forth by regional fisheries management organizations (RFMOs) or other international maritime regulatory bodies. These flags are called FOC because vessels carrying these flags are virtually guaranteed that, if they are to engage in illegal, unreported, and unregulated (IUU) fishing, they will face a significantly reduced likelihood of legal repercussions. Countries making these flags available have little incentive to carry out penalties should the vessels be caught violating the fisheries regulations set forth by national or international bodies. 

The International Transport Workers’ Federation (ITWF) is an international federation that represents 16 million transport workers in 700 unions hailing from 150 countries. The Federation has created a list of FOC countries basing it on their assessment of these countries’ performance according to different criteria (e.g., compliance with minimal regulations (e.g., at-sea labor regulations), registration procedures, absence of a “genuine link” between the beneficial owner of the vessel and the actual flag that vessel flies, and so on). The Federation lists 35 such countries as FOC countries. This ITWF list of FOC countries was used for the current analysis. The longlining activities of vessels flagged to these 35 FOC countries were identified by the ITWF to be used as a proxy for potential illegal longlining activity. 

Fishing data for these vessels were acquired from Global Fishing Watch (globalfishingwatch.org), an international initiative between Google, Oceana, and Skytruth that aims to provide “the world’s first global view of commercial fishing activities”. The website, which uses the Google platform, analyses billions of AIS signals received from fishing vessels worldwide. The data provided by Global Fishing Watch includes information on the type of gear used (e.g., longliner, trawler), and the flag that vessels carry, and are available at the one-half-degree by one-half-degree grid cell resolution. Data of longlining activities in 2016 were used because, at the time of this research, only data for this year were available in the format we needed for the subsequent analyses. 

We define FOC longlining activity risk spaces as areas where the fishing activities of longliners flagged to these 35 FOC countries have been recorded. The variable was dichotomously measured in terms of the presence (or absence) of FOC longline fishing activity within each grid cell in 2016. We acknowledge that this measure of potential illegal fishing activity is limited; however, it is one of the strongest proxy measures of illegal activity in the absence of actual data. Such measures have been used by the authors in the past, as mentioned earlier, including in the first study of illegal longline fishing and albatross extinction risk [15,30].

***Legal Longlining Activity.*** In addition to the information gathered through Global Fishing Watch on FOC-flagged vessels, we employed the same analytical strategies to gather data on the longlining activities of non-FOC-flagged vessels. After the data on all longline fishing activities were gathered (minus the activities carried out by FOC-flagged vessels), we converted the variable into a dichotomous measure in terms of the presence (or absence) of these activities within each grid cell. 

***Cumulative Risk Scores of CRAAVED Fish.*** As it pertains to IUU fishing, Petrossian and Clarke [37] created a list of 58 fish species that are known to be caught illegally internationally and assigned risk scores. From this list of 58 at-risk species, 28 fish species were identified as being caught with a longliner. The risk scores of each of the 28 species were used to calculate the *CRAAVED* fish risk space at the one-half-degree by one-half-degree grid cell level, similar to the calculations made to measure the dependent variable. 

Following this, the IUU risk scores assigned to each of these species were multiplied by the probability of occurrence of these species in each grid cell. The cumulative weighted risk score was, therefore, the sum of all these weighted risk scores. For example, if Species A’s probability in cell X is 0.5 and that species has an IUU risk score of 9, then the weighted risk score of the grid cell for Species A will be 9 × 0.5 = 4.5. If Species B’s probability in the same cell is 0.75 and that species has an IUU risk score of 9, then the weighted risk score of the grid cell for Species B will be 9 × 0.75 = 6.75. If no other species overlap within cell X, then the cumulative weighted risk of cell X to IUU fishing will be 4.5 + 6.75 = 11.25. Shapefiles of distribution ranges (and probabilities of occurrence) of the 28 fish species were extracted from the AquaMaps.com (accessed on 15 June 2021) database.

#### 2.2.3. Control Variables

***Sea Surface Salinity.*** This variable was obtained from NASA Earth Observations [44,45] and measures the saltiness of the ocean surface. The saltiness of ocean waters can impact where commercially sought out fish are found, thereby directly impacting where longliner vessels may be present. Data from 2015 (the latest year available) were downloaded, averaged, and joined to the existing one-half-degree by one-half-degree grid cell format for our analysis. 

***Sea Surface Temperature.*** Sea surface temperature could indirectly impact where longliner vessels are found at sea. Research has found that longline fishing effort intensity was highest between 16 and 19 °C isotherms [46]. These data were obtained from the NASA Earth Observations [47] for the year 2016, and were then averaged and joined to the grid cell shapefile.

### 2.3. Statistical Analysis

To test whether FOC longlining activity was spatially concentrated (H1), a Lorenz curve plot was created to graphically represent the distribution of this activity. More specifically, the cumulative percentage of FOC longliners across grid cells (1–159,087) was plotted against the cumulative percentage of grid cells to detect whether inequality is present. Coupled with a visual plot, the Gini coefficient [48] was then calculated based on the Lorenz curve using the formula shown in Equation (1), where *G* is the Gini coefficient; *A* is the area between the line of equality and the Lorenz curve; *B* is the area below the Lorenz curve. The Gini coefficient ranges from 0 to 1, with ‘0′ indicating perfect equality and ‘1′ indicating perfect inequality. If inequality was found, that would demonstrate that FOC longlining activity is highly concentrated.

(1) $$G=\frac{A}{A+B}$$

To test Hypothesis 2, LISA (local indicators of spatial autocorrelation) cluster maps were created for both the presence of FOC longliners and weighted CRAAVED fish risk spaces using GeoDa 1.2 software. LISA maps help to visualize where statistically significant hot and cold spots are clustered, along with spatial outliers—cold spots surrounded by hot spots and hot spots surrounded by cold spots [49]. Before creating the LISA cluster maps, spatial weights were created using the K-nearest neighbors of 6 so that all features have at least one neighbor. Statistical significance levels of *p* < 0.05 were used to identify clusters in the LISA maps. In addition, a Pearson correlation was run between the two independent variables, FOC longliners and weighted CRAAVED fish risk spaces. 

Hypothesis 3 was tested by building a multiple ordinary least-squares regression model. Specifically, the model was used to explain the average risk scores of albatrosses from the presence or absence of FOC-flagged vessels, the presence or absence of legal longliners, and the cumulative risk score of CRAAVED fish species when controlling for sea surface salinity and sea surface temperature. Multicollinearity tests were run between the independent variables, the skewness of all variables was tested, and variables were adjusted/log-transformed accordingly.

## 3. Results

**Figure 3:**
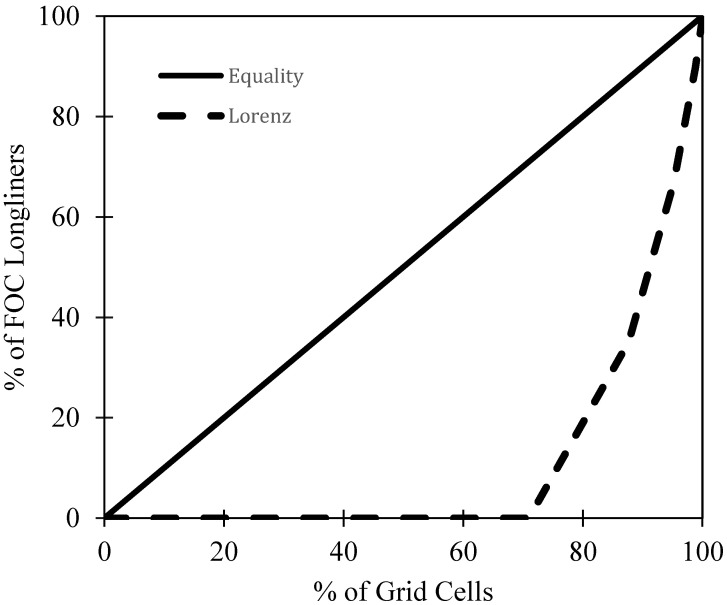
The inequality of FOC longliner presence across ocean grid cells in 2016. The Lorenz line (dotted) significantly deviates from the line of equality, demonstrating that FOC longliner presence is spatially concentrated in a small number of grid cells (Gini = 0.786). More specifically, 29% of at-sea grid cells experienced the presence of at least one FOC longliner, and this 29% accounted for 100% FOC longliner presence altogether.

FOC longline vessel presence was spatially concentrated according to Figure 3. The Lorenz line (dotted) significantly deviates from the line of equality, demonstrating that FOC longliner presence is spatially concentrated in a small number of grid cells (Gini = 0.786). More specifically, 29% of at-sea grid cells experienced the presence of at least one FOC longliner, and this 29% accounted for 100% of FOC longliner presence altogether. 

**Figure 4:**
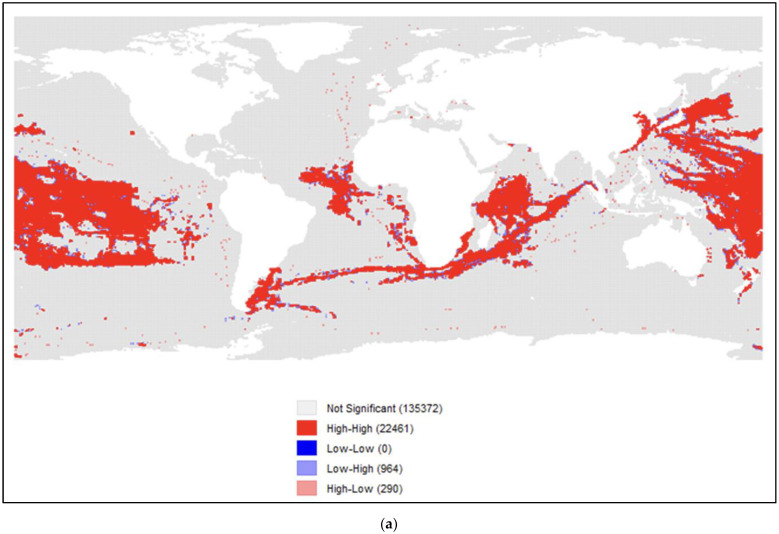

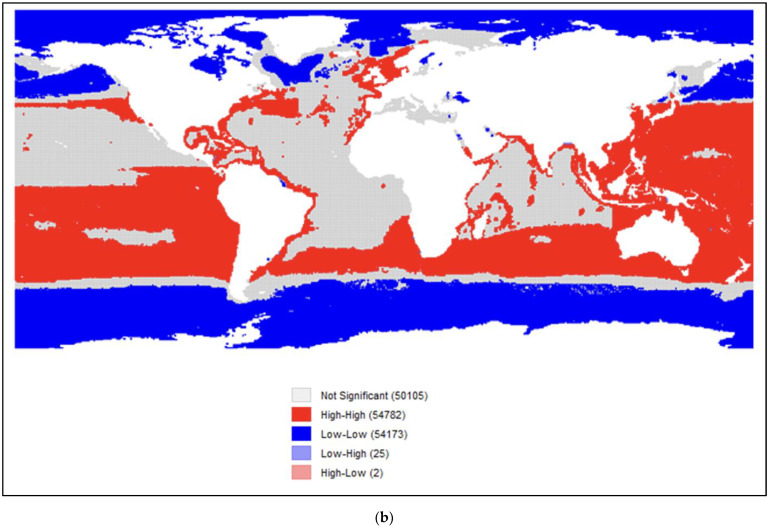
(**a**) A LISA cluster analysis of FOC longliners and (**b**) weighted CRAAVED fish presence. In (**a**) the LISA (local indicators of spatial autocorrelation) cluster map reveals a high positive autocorrelation (global Moran’s *I*: 0.82; *p* < 0.001), whereby approximately 14% of grid cells (high–high) experienced FOC longliner presence and were surrounded by grid cells experiencing similar phenomena. FOC longliner presence is significantly concentrated in certain areas, and this is hypothesized to be related to areas where there is a higher cumulative weighted risk of fish being CRAAVED. In (**b**), findings show a high positive autocorrelation (global Moran’s *I*: 0.98; *p* < 0.001), whereby approximately 34% of at-sea grid cells (high–high) experienced statistically significant clusters of CRAAVED fish presence. Comparing (**a**,**b**) with each other, it can be seen that FOC presence hot spots tend to overlap in CRAAVED fish hot spots, particularly in the Pacific Ocean near South America, Asia, Oceania, in the southern Atlantic Ocean, and in the Indian Ocean (*r* = 0.38; *p* < 0.001).

A visual representation of FOC longliner spatial concentration can be seen in Figure 4a. The LISA (local indicators of spatial autocorrelation) cluster map reveals a high positive autocorrelation (global Moran’s *I*: 0.82; *p* < 0.001), whereby approximately 14% of grid cells (high–high) experienced FOC longliner presence and were surrounded by grid cells experiencing similar phenomena. Most grid cells did not experience any kind of statistically significant clusters (135,372 cells, or 85%) of FOC longliner presence, though 290 cells (0.2%) experienced a high–low outlier value, whereby high-value cells were surrounded primarily by low-value cells. Altogether, longliner presence is significantly concentrated in certain areas, and this is hypothesized to be related to areas where there is a higher cumulative weighted risk of fish being CRAAVED (H2). To test this, a LISA cluster map (Figure 4b) was first generated to visualize the clustering of CRAAVED fish presence. Findings show a high positive autocorrelation (global Moran’s *I*: 0.98; *p* < 0.001), whereby approximately 34% of at-sea grid cells (high–high) experienced statistically significant clusters of CRAAVED fish presence. Similarly, approximately 34% of grid cells (low–low) experienced low CRAAVED fish presence and were similarly surrounded by cells with low CRAAVED fish presence. Comparing Figure 4a,b with each other, it can be seen that FOC presence hot spots tend to overlap in CRAAVED fish hot spots, particularly in the Pacific Ocean near South America, Asia, Oceania, in the southern Atlantic Ocean, and in the Indian Ocean (*r* = 0.38; *p* < 0.001). 

Table 1 and Table 2 provide descriptive statistics of the study variables and the results of the OLS regression model. According to Table 2, the overall results indicate that the variables collectively statistically significantly explain the average risk scores for albatrosses (F (5, 159,086) = 12,662 *p* < 0.01; R^2^ = 0.285). All the variables added statistically significantly to the model (*p* < 0.01). The average risk score of albatrosses is significantly higher in the areas where FOC-flagged or potentially illegal longline vessels are found. The average risk score of albatrosses is significantly lower in the areas where other longlining activity takes place. The cumulative risk score of CRAAVED fish species (i.e., their presence and risk of IUU fishing) can significantly explain the average risk score of albatrosses. In other words, the highest risk areas for CRAAVED fish species overlap with highest risk areas of albatrosses. The independent variable with the strongest beta value is the cumulative risk score of CRAAVED fish, followed by the presence of FOC-flagged longline vessels and the presence of legal longline vessels. 

## 4. Discussion

This study sets out to advance knowledge in this field in several ways. There was a significant gap in knowledge as to whether a spatial association existed between the conservation status of albatross species and illegal longline fishing activity. Whether the findings from one geographically limited study could be broadly generalized to a more global context was left unexplored. Shedding light on the impact of FOC longline fishing activities—used as a proxy measure of potentially illegal longlining activities—on albatross bycatch, this study expands on previous research in terms of its geographic scope and detail, as well as with the identification of illegal fishing vessel activities. The results of the current study demonstrate that spatial concentrations for potentially illegal longlining, commercially sought-out (or CRAAVED) fish, and albatross at sea-ranges do, in fact, exist, and that examining these concentrations from a crime pattern theory [36] approach helps to explain how perceived ‘victims’ (i.e., albatrosses) and ‘offenders’ (i.e., illegal longliners) converge in time and space. Through these analyses, it was possible to illustrate that albatrosses are, in fact, most at risk in areas where their at-sea ranges overlap with the presence of FOC-flagged longline fishing activities.

Specifically, this study demonstrated that FOC-flagged longlining activity is spatially concentrated and hot spots of this activity overlap with commercially sought-out fish hot spots. As expected, the highest areas for commercially sought-out fish also overlap with the highest risk areas for albatrosses. This is because albatrosses frequent areas with a high probability of occurrence of CRAAVED fish and longline vessels. Consequently, it was found that the cumulative risk score of albatrosses is significantly higher in areas where illegal longlining vessels are found. Illegal fishing vessels carrying a flag of convenience have been shown to disregard regulations and protocols set forth by RFMOs [16,17,18,19]. Such protocols may include the implementation of bycatch mitigation strategies [14] to reduce, for example, seabird deaths while trawling. Findings from this study suggest that FOC longline vessels are associated with an increased extinction risk for albatrosses, possibly because such bycatch mitigation strategies are seldomly used, or not used at all.

At the same time, legal longlining vessels are thought to be more likely to implement such measures, and our study demonstrates that their presence at sea is negatively related to the extinction risk of albatross species. This comes as a surprise, since prior research has found an association between seabird mortality and longline fishing at various scales [4,10,11,12,13,14]. These past findings are not consistent with our results potentially for several reasons. One, prior studies focused on all seabirds, and not exclusively on albatross species. Two, and more importantly, prior research did not differentiate between potentially bad actors—FOC longline vessels—and legal longline vessels. Longline vessels may not operate all in the same way, and it may be the case that FOC longline vessels disproportionately impact albatross species via bycatch. Third, many of these prior studies date back to the 1990s and early 2000s. It is possible that bycatch mitigation strategies are more commonly used by legal longlining vessels in 2016, the year of our analysis, than in the past. Should data become available, future research should investigate the relationship between FOC and non-FOC longlining vessels on the endangerment of all seabirds longitudinally to shed light on the activity of longlining vessels.

The novelty of this study is due, in part, to the application of crime pattern theory (CPT), an ecological theory of crime. This is only the second study that has explicitly applied CPT to a conservation problem caused by humans (see [50]). Importantly, this is the only study to date that has examined this problem at a high resolution at the global scale, and which subsequently has important policy implications. By examining the mobility patterns of offenders—in this case, fishing vessels—and victims, such as albatrosses, we can see patterned behavior in space much like that which is seen with traditional urban crimes. Future research should consider the application of CPT to IUU fishing or albatross bycatch research, considering the wealth of data at our disposal in this field. In comparison to traditional wildlife crime research where the geo-tagged location data of at-risk species and illegal actors is relatively rare, or even non-existent—especially at regional or global scales—geo-tagged fishing vessels and fish probability data are far easier to come by, as this study has demonstrated. 

### 4.1. Limitations

We use FOC as a proxy measure for illegal fishing, but it is important to note that FOC vessels are not necessarily operating illegally. We do so as the chances that a vessel flying an FOC is operating illegally is much higher than that of a vessel operating under a non-FOC flag. Due to the known association of FOC to IUU fishing, however, we make several recommendations. While suggesting the banning of FOC due to their known connections to IUU fishing (alongside other concerns) is unnecessary and, generally speaking, unfeasible, flag state responsibility is an area of mitigation warranting further attention. The lack of flag state responsibility has been argued to be an element leading to the emergence of an FOC market in which certain desirable flags also facilitate IUU fishing. Other means by which to address FOC include closing open registry systems to fishing vessels; banning, by coastal states and RFMOs, the use of FOC by fishing vessels authorized to fish within their EEZs and management areas; flag states, coastal states, and RFMOs maintaining a publicly available list of authorized vessels and forms of access agreements; and countries maintaining a public register of their entire fishing fleets, including foreign-flagged vessels owned by their nationals. Details on these recommendations and others can be found in Petrossian et al. (see [19]).

In this paper, we overlayed vessel activity with the cumulative risk score of all 22 albatross species as measured by their IUCN Red List status. While it is known that some species meet Red List criteria because of their small populations, which can be related to inherent demographic characteristics or because of threats apart from fishing, most of the 22 albatross species are threatened by fishing as the leading threat to their survival. In fact, for 17 of the 22 species, fishing is the leading threat according to the IUCN Red List information from 2016, the year of the current analysis. Further, of the seven endangered or critically endangered albatross species, fishing is the leading threat (see Appendix A). 

### 4.2. Policy Implications

There are over 130 agreements, regulations, and legislations on the reduction of bycatch around the world, spanning from net mesh measures, fishing area designations, rules for the discarding of fish, the use of bycatch mitigation measures, and various others [51]. Among these, for example, is the Agreement on the Conservation of Albatrosses and Petrels, a major conservation instrument for albatrosses and petrels and a forum where bycatch and other fisheries related threats are discussed. However, to have their intended impact, these policies need to be implemented, monitored, and enforced by regional fisheries management organizations (RFMOs) and other national and international bodies. The input of these international organizations, especially that of RFMOs, is vital considering that the high seas fall within their management realm and are not the responsibility of states. 

According to BirdLife International, there are five RFMOs whose geographic scope overlaps with albatross distributions [46]. These include the Commission for Conservation of Southern Bluefin Tuna (CCSBT), the Western and Central Pacific Fisheries Commission (WCPFC), the Indian Ocean Tuna Commission (IOTC), the International Commission for the Conservation of Atlantic Tunas (ICCAT), and the Commission for the Conservation of Antarctic Marine Living Resources (CCAMLR) [10]. The use of bycatch mitigation measures by CCAMLR, back in 2004, had reduced their seabird bycatch by over 99%, indicating the importance and value of enforcing the use of such measures [10]. However, over 80% of global albatross distribution is found outside of CCAMLR waters, overlapping mainly with tuna and swordfish fisheries, which are managed by the world’s five tuna commissions [38]. This highlights both the need for utilizing bycatch mitigation strategies due to the demonstrated efficacy and need for enforcing these strategies in the RFMOs overlapping with albatross at-sea ranges. It is important to point out that while regional fisheries management organizations are the inter-governmental bodies solely responsible for managing the fisheries beyond the jurisdiction of coastal countries, they are limited in their ability to provide governance in the high seas. It is, therefore, vital that the RFMOs implement more rigorous strategies to strengthen their management and enforcement capacity. 

Unfortunately, high levels of non-compliance with bycatch mitigation regulations remain a problem. The idea is supported that non-compliance with RFMO seabird conservation strategies in authorized vessels should be recognized as a form of illegal, unreported, and unregulated fishing [40]. Other means to both enforce and monitor the use of bycatch mitigation strategies include the more regular use of onboard observers and the enforcement of penalties for non-compliance. 

### 4.3. Reducing IUU Fishing

On a more global level, efforts to reduce IUU fishing would positively impact albatross bycatch reduction. Petrossian [29] has previously applied situational crime prevention strategies (a family of crime reduction practices emerging from the field of crime science) to examine and outline prevention mechanisms for IUU fishing on a global scale. These strategies include increasing patrol surveillance using onboard observers (or using electronic monitoring systems), strengthening trade regulations on certain species of fish stimulating IUU fishing, and addressing the issue of ports of convenience that enable offloading illegally obtained fish. The results of the current study will help focus such measures to areas within the RFMOs that display both high levels of FOC-flagged fishing vessel efforts and high at-risk albatross presence. 

**Figure 5:**
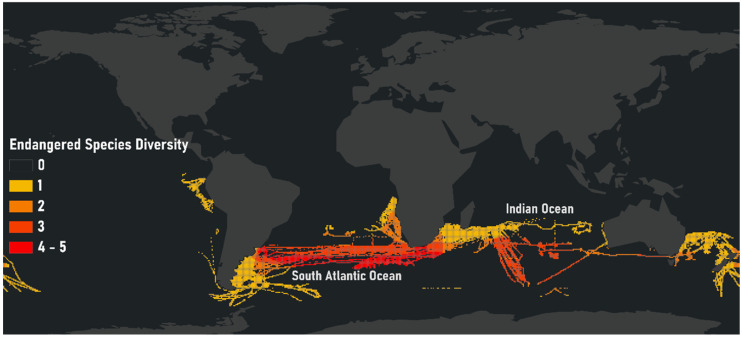
Endangered albatross species’ diversity within FOC longliner Areas. FOC longliners were present in 46,165 grid cells of the 159,087 at-sea grid cells in 2016. Of these 46,165 grid cells, 18% (8483 grid cells) overlapped with the ranges of at least one threatened albatross species. Such at-risk areas for IUU fishing should be the focus of prevention and bycatch mitigation strategies to reduce the mortality rates of protected albatross species.

To this end, of the 159,087 at-sea grid cells, FOC longliners were present in 46,165 grid cells in 2016. However, not all cells overlap with the distributions of endangered albatrosses. Of these 46,165 grid cells, 18% (8483 grid cells) overlapped with the ranges of at least one threatened albatross species (Figure 5). Such at-risk areas for IUU fishing should be the focus of prevention and bycatch mitigation strategies to reduce mortality rates of protected albatross species.

## 5. Conclusions

Wild animals and plants provide vital ecosystem services, such as flood retention, carbon storage, and water filtering, playing an integral role in retaining the ecological and biological balance on our planet. Nevertheless, the last several decades have seen unprecedented increases in crimes against wildlife, affecting thousands of genera and species. The impacts of these crimes can potentially lead to irreversible damages for some species, leading to their possible extinction. For other species, the impact is gradual and consistent. Among the latter are the albatrosses, some of which are now critically endangered and are facing the risk of extinction. Albatrosses already suffer from a large range of other threats both on land and at sea, such as the loss or degradation of their nesting habitat, various diseases, the impacts of invasive species, and pollution and climate change [52]. Increasing efforts on addressing illegal fishing activities, especially those occurring within the at-sea ranges and areas that overlap with high-risk albatross species is, therefore, vital and urgently needed more so now than ever before. Such efforts should be swift, concerted, and focused, so that they will not only reduce the illegal fishing and overexploitation of many vulnerable marine species, but also have an impact on the preservation of the most iconic bird species that roam the oceans. 

## Figures and Tables

**Table 1:** Descriptive statistics of study variables. Basic descriptive statistics of the outcome variable (average albatross risk score, calculated as the cumulative risk score of all the albatrosses within a grid cell and based on their IUCN Red List status divided by the number of albatrosses within the given grid cell); three independent variables (cumulative risk score of CRAAVED fish, measured using the risk scores created by Petrossian and Clarke [37]; FOC-flagged longline vessel presence (yes = 1); and other longline vessel presence (yes = 1)); and two control variables (sea surface salinity and temperature).

Variable	N	Min	Max	Mean	SD
*Outcome Measure*					
Average albatross risk score (DV)	159,087	0	5	0.94	0.87
*Independent Variables*					
Cumulative risk score of CRAAVED fish	159,087	0	155	36.24	24.99
FOC-flagged longliners	159,087	0	1	--	0.454
Other longliners	159,087	0	1	--	0.499
*Control Variables*					
Sea surface salinity (SSS)	159,087	0	0.04	0.03	0.01
Sea surface temperature (SST)	159,087	−1.18	30.83	14.70	11.17

**Table 2:** Explaining the average risk score of albatrosses. Ordinary least-squares regression analysis examining the variation of the average risk score of albatrosses and the contribution of the independent variables. The overall model can explain 28% of the variance in the outcome variable. The average risk score of albatrosses is significantly higher in the areas where FOC-flagged vessels are found and significantly lower in the areas where other longlining activities take place. The presence and risk of CRAAVED fish to IUU fishing species can explain the average risk scores of albatrosses.

	B	SEB	β
Intercept	0.073	0.005	-
FOC longliners present	0.200	0.006	−0.104 **
Legal longliners present	−0.093	0.006	−0.053 **
Cumulative risk score of CRAAVED fish	0.011	0.000	−0.324 **
SSS	40.58	0.182	−0.557 **
Hurricane + Cyclone	−0.023	0.011	−0.004 *
SST	−0.053	0.000	−0.679 **

Note: R^2^ = 0.285; adjusted R^2^ = 0.285; F (5, 159,086) = 12,662, *p* < 0.01; ** *p* < 0.001; * *p* < 0.05.

## Data Availability

Publicly available datasets were analyzed in this study. This data can be found here: http://datazone.birdlife.org (accessed on 2 January 2021) and other sources described in Materials and Methods. Excel datasets and shapefiles used to generate the maps are available upon request.

## Abbreviations

CPT	Crime pattern theory
CRAAVED	Concealable, removable, accessible, abundant, valuable, enjoyable, disposable
FOC	Flags of convenience
ITWF	International Transport Workers’ Federation
IUCN	International Union for Conservation of Nature
IUU	Illegal, unreported, and unregulated fishing
RFMO	Regional fisheries management organization

## Appendix A. Data on 22 Albatross Species

**Table A1:** List of 22 albatross species and their 2016 IUCN Red List status as NT = Near Threatened, VU = Vulnerable, EN = Endangered, and CR = Critically Endangered (Source: Birdlife International, http://datazone.birdlife.org/species/spcredcat, accessed on 15 June 2021). Also presented the impact severity of their major threats, including fishing, domestic cats, and rats. Scores represent causing or likely to cause (4) very rapid declines (>30% over 10 years or three generations), (3) rapid declines (20–30% over 10 years or three generations), (2) relatively slow but significant declines (<20% over 10 years or three generations), (1) fluctuations, and (0) negligible or no declines (modified from source: Birdlife International, http://datazone.birdlife.org/species/spcthreat, accessed on 15 June 2021).

Common Name	Latin Name	IUCN Red List Status	Impact Severity		
		2016	Fishing	Domestic Cats	Rats
Amsterdam Albatross	*Diomedea amsterdamensis*	CR	4	1	0
Tristan Albatross	*Diomedea dabbenena*	CR	4	0	3
Waved Albatross	*Phoebastria irrorata*	CR	3	0	0
Atlantic Yellow-nosed Albatross	*Thalassarche chlororhynchos*	EN	3	0	0
Black-browed Albatross	*Thalassarche melanophrys*	NT	4	-	0
Indian Yellow-nosed Albatross	*Thalassarche carteri*	EN	4	0	0
Northern Royal Albatross	*Diomedea sanfordi*	EN	2	1	-
Sooty Albatross	*Phoebetria fusca*	EN	3	2	2
Antipodean Albatross	*Diomedea antipodensis*	VU	-	-	0
Black-footed Albatross	*Phoebastria nigripes*	NT	3	1	2
Campbell Albatross	*Thalassarche impavida*	VU	0	0	0
Chatham Albatross	*Thalassarche eremita*	VU	0	0	0
Grey-headed Albatross	*Thalassarche chrysostoma*	VU	4	0	0
Salvin’s Albatross	*Thalassarche salvini*	VU	1	0	0
Short-tailed Albatross	*Phoebastria albatrus*	VU	2	0	2
Southern Royal Albatross	*Diomedea epomophora*	VU	3	0	0
Wandering Albatross	*Diomedea exulans*	VU	4	2	0
Buller’s Albatross	*Thalassarche bulleri*	NT	0	0	0
Laysan Albatross	*Phoebastria immutabilis*	NT	3	0	1
Light-mantled Albatross	*Phoebetria palpebrata*	NT	3	-	0
Shy Albatross	*Thalassarche cauta*	NT	-	0	0
White-capped Albatross	*Thalassarche steadi*	NT	2	0	0

## References

[1] Tuck G.N., Polacheck T., Bulman C.M. (2003). Spatio-temporal trends of longline fishing effort in the Southern Ocean and implications for seabird bycatch. Biol. Conserv..

[2] Gandini P., Frere E. (2003). Spatial and temporal patterns in the by-catch of seabirds in the Argentinian long-line fishery. Fish. Bull..

[3] Croxall J.P., Butchart S.H.M., Lascelles B., Stattersfield A.J., Sullivan B., Symes A., Taylor P. (2012). Seabird conservation status, threats and priority actions: A global assessment. Bird Conserv. Int..

[4] Huang H. (2011). By-catch of high sea long-line fisheries and measures taken by Taiwan: Actions and challenges. Mar. Policy.

[5] Gilman E. (2004). References on Seabird by-Catch in Long-Line Fisheries.

[6] Anderson O.R.J., Small C.J., Croxall J.P., Dunn E.K., Sullivan B.J., Yates O., Black A. (2011). Global seabird by-catch in long-line fisheries. Endanger. Species Res..

[7] Gales R., Brothers N., Reid T. (1998). Seabird mortality in the Japanese tuna long-line fishery around Australia, 1988–1995. Biol. Conserv..

[8] Gilman E. (2003). Marine Matters Seabird Mortality in North Pacific Longline Fisheries. Endanger. Species.

[9] Lewison R.L., Crowder L.B., Read A.J., Freeman S.A. (2004). Understanding impacts of fisheries by-catch on marine megafauna. Trends Ecol. Evol..

[10] Small C.J. (2005). Regional Fisheries Management Organizations: Their Duties and Performance in Reducing by-Catch of Albatrosses and Other Species.

[11] Nel D.C., Taylor F.E. (2003). Globally Threatened Seabirds at Risk from Long-Line Fishing: International Conservation Re-Sponsibilities.

[12] Weimerskirch H., Brothers N., Jouventin P. (1997). Population dynamics of wandering albatross *Diomedea exulans* and Amsterdam albatross *D. amsterdamensis* in the Indian Ocean and their relationships with long-line fisheries: Conservation implications. Biol. Conserv..

[13] Croxall J.P., Prince P.A. (1996). Potential interactions between wandering albatrosses and long-line fisheries for Patagonian toothfish at South Georgia. CCAMLR Sci..

[14] Pott C., Wiedenfeld D.A. (2017). Information gaps limit our understanding of seabird bycatch in global fisheries. Biol. Conserv..

[15] Petrossian G.A., de By R., Clarke R.V. (2016). Illegal long-line fishing and albatross declines. Oryx.

[16] EJF (2009). Lowering the Flag—Ending the Use of Flags of Convenience by Pirate Fishing Vessels.

[17] UNODC (United Nations Office on Drugs and Crime) (2011). Transnational Organized Crime in The Fishing Industry.

[18] George R. (2011). Flying the Flag, Fleeing the State. The New York Times.

[19] Petrossian G.A., Sosnowski M., Miller D., Rouzbahani D. (2020). Flags for sale: An empirical assessment of flag of convenience desirability to foreign vessels. Mar. Policy.

[20] Wortley R., Mazerolle L. (2008). Environmental Criminology and Crime Analysis.

[21] Andresen M.A., Malleson N. (2013). Crime seasonality and its variations across space. Appl. Geogr..

[22] Ratcliffe J.H., Rengert G.F. (2008). Near-Repeat Patterns in Philadelphia Shootings. Secur. J..

[23] Farrell G., Pease K. (2017). Preventing Repeat and Near Repeat Crime Concentrations. Handbook of Crime Prevention and Community Safety.

[24] Fagan A.A., Mazerolle P. (2008). Repeat Offending and Repeat Victimization: Assessing Similarities and Differences in Psychosocial Risk Factors. Crime Delinq..

[25] Lemieux A.M., Clarke R.V. (2009). The International Ban on Ivory Sales and its Effects on Elephant Poaching in Africa. Br. J. Criminol..

[26] Pires S.F., Clarke R.V. (2011). Sequential Foraging, Itinerant Fences and Parrot Poaching in Bolivia. Br. J. Criminol..

[27] Pires S., Clarke R.V. (2011). Are Parrots CRAVED? An Analysis of Parrot Poaching in Mexico. J. Res. Crime Delinq..

[28] Pires S.F., Olah G., Nandika D., Agustina D., Heinsohn R. (2021). What drives the illegal parrot trade? Applying a criminological model to market and seizure data in Indonesia. Biol. Conserv..

[29] Petrossian G.A. (2015). Preventing illegal, unreported and unregulated (IUU) fishing: A situational approach. Biol. Conserv..

[30] Petrossian G.A. (2018). A micro-spatial analysis of opportunities for IUU fishing in 23 Western African countries. Biol. Conserv..

[31] Weekers D.P., Zahnow R. (2018). Risky facilities: Analysis of illegal recreational fishing in the Great Barrier Reef Marine Park, Australia. Aust. N. Z. J. Criminol..

[32] Weekers D., Petrossian G., Thiault L. (2021). Illegal fishing and compliance management in marine protected areas: A situational approach. Crime Sci..

[33] Skidmore A. (2021). Using crime script analysis to elucidate the details of Amur tiger poaching in the Russian Far East. Crime Sci..

[34] Viollaz J.S., Thompson S.T., Petrossian G.A. (2021). When Human–Wildlife Conflict Turns Deadly: Comparing the Situational Factors That Drive Retaliatory Leopard Killings in South Africa. Animals.

[35] Marteache N., Pires S.F. (2019). Choice Structuring Properties of Natural Resource Theft: An Examination of Redwood Burl Poaching. Deviant Behav..

[36] Brantingham P.J., Brantingham P.L. (1993). Environment, routine and situation: Toward a pattern theory of crime. Adv. Criminol. Theory.

[37] Petrossian G.A., Clarke R.V. (2013). Explaining and Controlling Illegal Commercial Fishing: An Application of the CRAVED Theft Model. Br. J. Criminol..

[38] Birdlife International (2022). Birdlife is Working with Regional Fisheries Management Organizations to Reduce Albatross Declines. http://datazone.birdlife.org/home.

[39] IUCN (2016). The IUCN Red List of Threatened Species.

[40] Seabird Bycatch Working Group (2019). Workshop. https://ebrc.org/wp-content/uploads/2019/08/NSTC-Workshop-Goals.pdf.

[41] Warner-Kramer D. (2004). Control begins at home: Tackling flags of convenience and IUU fishing. Gold. Gate UL Rev..

[42] Ford J.H., Wilcox C. (2019). Shedding light on the dark side of maritime trade–A new approach for identifying countries as flags of convenience. Mar. Policy.

[43] Miller D.D., Sumaila U.R. (2014). Flag use behavior and IUU activity within the international fishing fleet: Refining definitions and identifying areas of concern. Mar. Policy.

[44] NASA Earth Observations Sea Surface Salinity. https://neo.gsfc.nasa.gov/view.php?datasetId=AQUARIUS_SSS_M.

[45] National Centers for Environmental Information. https://www.ncdc.noaa.gov/sotc/tropical-cyclones/202008.

[46] Kroodsma D.A., Mayorga J., Hochberg T., Miller N.A., Boerder K., Ferretti F., Wilson A., Bergman B., White T.D., Block B.A. (2018). Tracking the global footprint of fisheries. Science.

[47] NASA Earth Observations (2021). Sea Surface Temperature. https://neo.gsfc.nasa.gov/view.php?datasetId=MYD28M.

[48] Gini C. (1921). Measurement of Inequality of Incomes. Econ. J..

[49] Anselin L. (1995). Local Indicators of Spatial Association—LISA. Geogr. Anal..

[50] Kurland J., Pires S.F., Marteache N. (2018). The spatial pattern of redwood burl poaching and implications for preven-tion. For. Policy Econ..

[51] Kibel P. (2018). New Technology Reduces Harm to Marine Species. https://theecologist.org/2018/oct/19/new-technology-reduces-harm-marine-species.

[52] Phillips R., Gales R., Baker G., Double M., Favero M., Quintana F., Tasker M., Weimerskirch H., Uhart M., Wolfaardt A. (2016). The conservation status and priorities for albatrosses and large petrels. Biol. Conserv..

